# Probiotics/synbiotics supplementation reduce the infection incidence in patients undergoing resection for colorectal cancer: an umbrella review

**DOI:** 10.3389/fmicb.2025.1635409

**Published:** 2025-09-11

**Authors:** Shilin Gao, Xi Liao, Yuhua He, Jie Yang

**Affiliations:** Colorectal Cancer Center, West China Hospital, Sichuan University/West China School of Nursing, Sichuan University, Chengdu, Sichuan, China

**Keywords:** probiotic, synbiotic, prebiotic, colorectal cancer, gut microbiota, umbrella review

## Abstract

**Objectives:**

This study reviews meta-analyses of perioperative supplementation with probiotics/synbiotics in colorectal cancer (CRC), systematically assessing the quality of meta-analyses and synthesizing study results to provide robust evidence.

**Methods:**

This review was conducted in accordance with the Preferred Reporting Items for Systematic Reviews and Meta-Analyses (PRISMA) statement. The search was conducted by two authors in four databases, PubMed, CENTRAL, EMBASE and Web of Science, up until August 3rd, 2025, and conducted independent assessments of the methodological quality of the meta-analysis via A Measure Tool to Assess Systematic Reviews (AMSTAR) 2.

**Results:**

A total of 11 meta-analyses were included in this umbrella review. 3 meta-analyses rated “Critically low” shared ≥3 critical flaws and 2 high-rated reviews adhered to ≥80% AMSTAR 2 criteria. Compared with the control group, the probiotic/synbiotic group presented lower incidence rates of overall infections (OR 0.49, 95% CI: 0.43, 0.56; *P* < 0. 001, I^2^ = 6%), surgical site infections (OR 0.58, 95% CI: 0.50, 0.67; *P* < 0.00001, I^2^ = 0%), urinary tract infections (OR 0.39, 95% CI: 0.27, 0.54; *P* < 0.00001, I^2^ = 0%), and pneumonia infections (OR 0.34, 95% CI: 0.26, 0.45; *P* < 0.00001, I^2^ = 0%), and diarrhea incidence (OR 0.41, 95% CI: 0.34, 0.51; *P* < 0.00001, I^2^ = 0%).

**Conclusion:**

According to the results of our analyses, perioperative probiotic/synbiotic supplementation in CRC patients is associated with a reduced incidence of overall infections, surgical site infections, urinary tract infections, pneumonia infections, and diarrhea.

**Systematic review registration:**

https://www.crd.york.ac.uk/prospero/, identifier CRD42024619853.

## Introduction

Colorectal cancer is the third most common cancer and the second leading cause of cancer death worldwide, and is a major contributor to morbidity and mortality in the global population (Siegel et al., 2023). Patients with CRC can experience certain intestinal microecological disturbances of their own, such as a significant enrichment of pathogenic bacteria such as *Clostridium nucleatum*, *Streptococcus anaerogenes*, *Aeromonas riceii*, and *Staphylococcus stomatitis*, and a decrease in the composition of Thermophilic *Streptococcus*, *Lactobacillus fowleri*, *Streptococcus maltophagus*, *Clostridium butyricum* and *Streptococcus salivarius*, which constitute a decreased proportion, etc., and this imbalance is exacerbated by preoperative mechanical bowel preparation and surgery (Jin et al., 2019; Weaver et al., 2024; Yu et al., 2017). After CRC surgery, the number of Bifidobacteria and the Shannon index decreased, whereas the proportions of Enterococci, staphylococci, and *Pseudomonas* increased. This change in the commensal-pathogenic bacterial balance after surgery may lead to complications such as surgical site infection (SSI) (Masheghati et al., 2024). Among these, SSI is significantly associated with recurrence and survival in CRC patients, and its incidence should be minimized to improve surgical and long-term oncological outcomes (Chen et al., 2021).

Recent studies have shown that the gut microbiome is often associated with cancer therapies (including surgical, chemical, radiation, and immune therapies), therapeutic effectiveness, and side effects (Dai et al., 2024; Zhou et al., 2024). Previous studies have confirmed that probiotic supplementation during the perioperative period of CRC produces significant clinical benefits, reducing the incidence of intestinal obstruction, peritoneal effusion, diarrhea, sepsis, pneumonia and SSI in CRC patients (Araújo et al., 2023). In addition, probiotics can improve the intestinal microenvironment and promote the repair and regeneration of the intestinal mucosa, thus accelerating the recovery of postoperative intestinal function, which can help reduce the incidence of postoperative intestinal paralysis, intestinal obstruction, and other complications (Chen et al., 2022; Liu et al., 2016). CRC patients may suffer from dyspepsia, nausea, diarrhea and other discomfort during the perioperative period, and probiotic supplementation can help alleviate these discomforts by adjusting the balance of the intestinal flora and improving the digestive function to alleviate these discomfort symptoms (Kasatpibal et al., 2017; Xu et al., 2019). The probiotic groups most widely used in the perioperative period of CRC are *Lactobacillus* and *Bifidobacterium*, which play important roles in promoting food absorption, enhancing host resistance to infection, strengthening the intestinal immune system, and regulating host metabolism (Eslami et al., 2019).

Although probiotic/synbiotic supplementation in the perioperative period of CRC has received increasing attention and numerous meta-analyses have been conducted to evaluate it, the results are inconsistent (Araújo et al., 2023; Kothari et al., 2019). Umbrella reviews represent the pinnacle of evidence-based medicine, systematically assessing the quality of meta-analyses and synthesizing relevant findings to provide reliable evidence (Tang et al., 2024). Therefore, to provide new perspectives for clinical practice, the present study is an umbrella review of meta-analyses on perioperative probiotic/synbiotic supplementation in CRC.

## Methods

Our work has been reported in line with PRISMA (Page et al., 2021) and AMSTAR 2 (Shea et al., 2017) Guidelines.

### Search strategy

Two authors conducted a comprehensive literature search of PubMed, Cochrane Central Register of Controlled Trials, EMBASE and Web of Science from inception to August 3rd, 2025 via a combination of Medical Subject Headings (MeSH) and free words. The keywords selected were “probiotics,” “symbiotic,” “meta” and “colorectal cancer,” etc. The search strategy was adapted to the characteristics of each database. (Supplementary Appendix 1 shows an example search strategy for the PubMed database). In addition, references to the included articles were manually searched for additional studies. All studies were imported into EndNote (version X9.2), where they were deduplicated. When differences of opinion were encountered, a third author was involved in the discussion until a consensus was reached.

### Eligibility criteria

To identify relevant meta-analysis studies, we outlined inclusion criteria according to the reporting structure of populations, interventions, comparisons, outcomes and study designs (PICOs): (1) any meta-analysis comparing perioperative probiotic/synbiotic preparations with placebo or standard care in elective colorectal cancer surgery; and (2) a meta-analysis including only Randomized controlled trials (RCTs). The exclusion criteria were as follows (1) no clinical outcomes associated with the use of probiotics/synbiotics were reported, and (2) systematic reviews without meta-analyses.

### Data extraction

**FIGURE 1:**
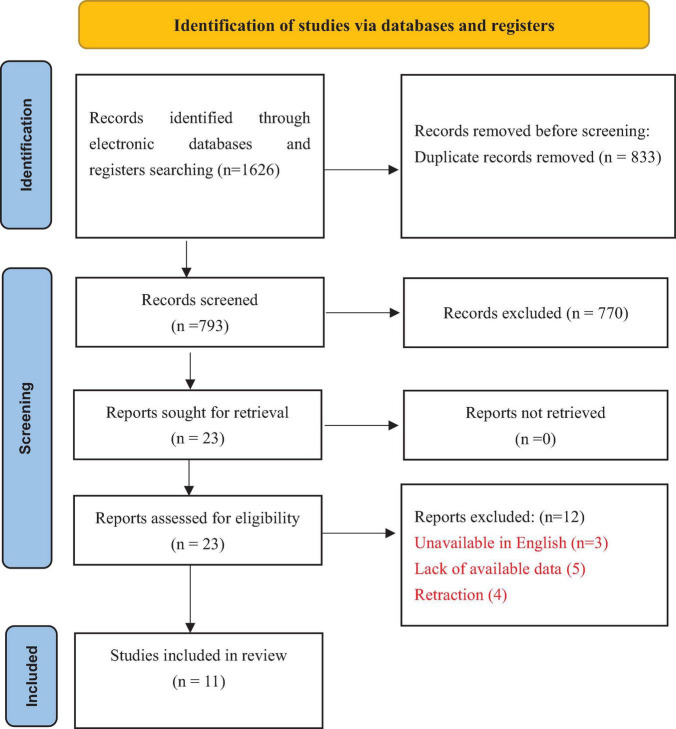
The PRISMA flow diagram to show study selection.

We extracted the characteristics of all included meta-analysis studies via a predesigned custom Microsoft Excel spreadsheet, and the selection process is summarized in Figure 1. These data included the following details: (1) Study characteristics: author, publication date, number of studies and participants, outcome and AMSTAR 2 quality. (2) Primary outcomes: overall infection incidence and surgical site infections; secondary outcomes: urinary tract infection, pneumonia infection, diarrhea incidence, effect of perioperative administration of probiotics/synbiotics on surgical site infections, and effect of preoperative/preoperative and postoperative administration of probiotics on overall infection complications.

### Quality assessment

Two reviewers independently assessed the quality of the meta-analysis methodology via AMSTAR 2, with results expressed as “high,” “medium,” “low” or “critically low.” When disagreements arose, they were discussed with a third author to reach a consensus.

### Data analysis

Statistical analysis was performed via RevMan 5.4. The results of the meta-analyses were pooled and expressed as the mean difference (MD) or odds ratio (OR) with the corresponding 95% CIs. For dichotomous variables, we used the Mantel-Haenszel method to run fixed and random object models. The inverse variance method was used for continuous variables. If I^2^ was ≥50%, there was significant heterogeneity, and a random effects model was used to combine the effect sizes. If I^2^ is <50%, the heterogeneity is small, and the fixed-effects model can be used to combine the effect size. The test level was bilateral (α = 0.05).

## Results

### Search results

In accordance with the search strategy, the two authors initially retrieved a total of 1,626 articles, of which 833 were excluded because of duplication and 770 were excluded because the titles and abstracts were read. The study selection flow and the reasons for exclusion are summarized in Figure 1. Finally, 11 studies were included (Amitay et al., 2020; An et al., 2022; Chen et al., 2022, 2024; de Andrade Calaça et al., 2017; Jiang and Ren, 2024; Liu et al., 2016, 2017; Ouyang et al., 2019; Paterson et al., 2025; Persson et al., 2024; Veziant et al., 2022; Wu et al., 2018; Zeng et al., 2021). Figure 1 illustrates the literature screening process.

### Study characteristics

All included studies were published between 2017 and 2025. Each meta-analysis included 7–28 RCTs; two studies did not report the population size (Amitay et al., 2020; Chen et al., 2024), and the remaining studies, Paterson et al. (2025) included a maximum of 2,686 participants. Notably, 3 meta-analyses rated “Critically low” (de Andrade Calaça et al., 2017; Liu et al., 2017; Ouyang et al., 2019) shared ≥3 critical flaws. Such as, inadequate gray literature retrieval (potentially missing negative results); Failure to assess RoB in primary RCTs (undermining heterogeneity interpretation); Lack of excluded study documentation (reducing reproducibility). These limitations collectively diminish confidence in their pooled effects. Conversely, high-rated reviews (Veziant et al., 2022; Wu et al., 2018) adhered to ≥80% AMSTAR 2 criteria. Table 1 shows the basic information of all included studies.

**TABLE 1:** Basic information of the included studies.

References	No. of studies/participants, *n*	Duration of administration	Outcome	AMSTAR 2 quality
Amitay et al., 2020	11/NR	During chemotherapy to 2 years after end of CRC treatment	Septicemia and sepsis, infection incidence, diarrhea incidence, LOS, return to normal gut function and first defecation, days of antibiotics use	Medium
An et al., 2022	19/1763	15 days before surgery to 30 days after surgery	Overall postoperative infection complication, probiotics-related adverse events, overall postoperative complications, LOS, quality of Life	Medium
Chen et al., 2022	14/1566	8 days before surgery to 15 days after surgery	Postoperative infection complications	Low
de Andrade Calaça et al., 2017	7/821	12 days before surgery to 12 days after surgery	Postoperative infection complications, detection of bacteria in blood	Critically low
Liu et al., 2017	9/1146	11 days before surgery to 15 days after surgery	SSI, urinary tract infection, pneumonia, bacteremia, bacterial translocation, anastomotic leakage	Critically low
Ouyang et al., 2019	13/1186	8 days before surgery to 15 days after surgery	Overall infection rate, pneumonia, the first flatus time, urinary tract infection, anastomotic leakage, and duration of postoperative pyrexia	Critically low
Persson et al., 2024	10/1276	1 days before surgery to 30 days after surgery	Diarrhea, SSI, urinary infection, pulmonary infection, abdominal distention, duration of postoperative pyrexia days, LOS, time to first defecation, and time to first solid diet	Medium
Veziant et al., 2022	21/1961	5 days before surgery to 1 year after surgery	SSI, pulmonary infections, urinary infections, anastomotic leaks, wound infections, postoperative infections	High
Wu et al., 2018	34/2634	9 days before surgery to 6 months after surgery	SSI, postoperative infections, safety, cost-effectiveness, quality of life, intensive care unit stay and hospital stay and promoted earlier first defecation and first bowel movement	High
Zeng et al., 2021	19/1975	9 days before surgery to 15 days after surgery	SSI, inflammatory factors, intestinal dysbiosis, infection complications, and systemic symptoms, systemic inflammatory response syndrome incidence, celiac infections	Medium
Paterson et al., 2025	28/2686	9 days before surgery to 30 days after surgery	Overall postoperative infection, SSI, urinary infection, pulmonary infection	Low

NR, not reported; LOS, length of stay; SSI, surgical site infection.

**FIGURE 2:**
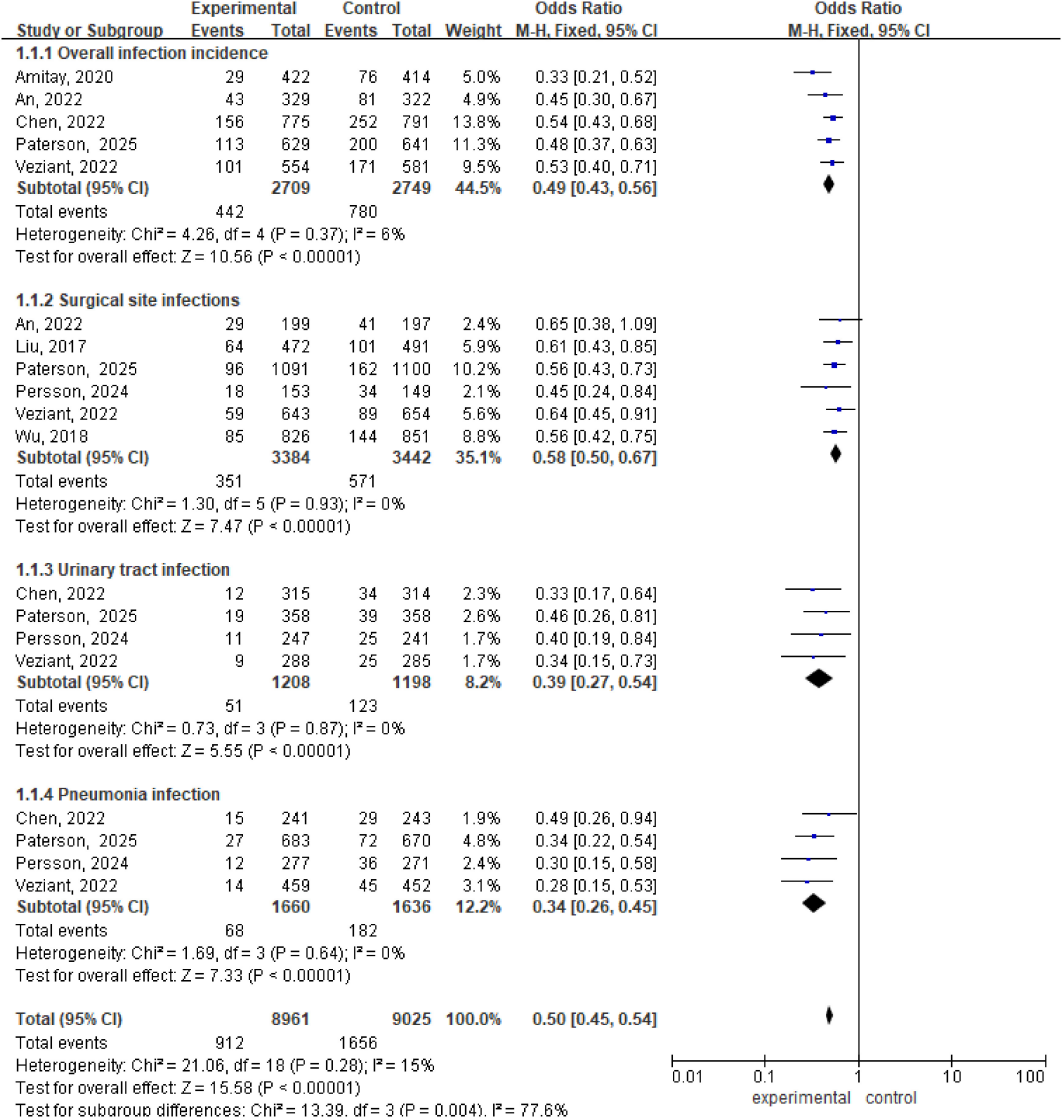
Forest plot of overall infection incidence, surgical site infections, urinary tract infection, and pneumonia infection.

Analysis via a fixed effects model revealed significant lower in the incidence of overall infection incidence between the intervention and control groups (OR 0.49, 95% CI: 0.43, 0.56; *P* < 0.001, I^2^ = 6%), surgical site infections (OR 0.58, 95% CI: 0.50, 0.67; *P* < 0.00001, I^2^ = 0%), urinary tract infections (OR 0.39, 95% CI: 0.27, 0.54; *P* < 0.00001, I^2^ = 0%), and pneumonia infections (OR 0.34, 95% CI: 0.26, 0.45; *P* < 0.00001, I^2^ = 0%) (Figure 2).

**FIGURE 3:**
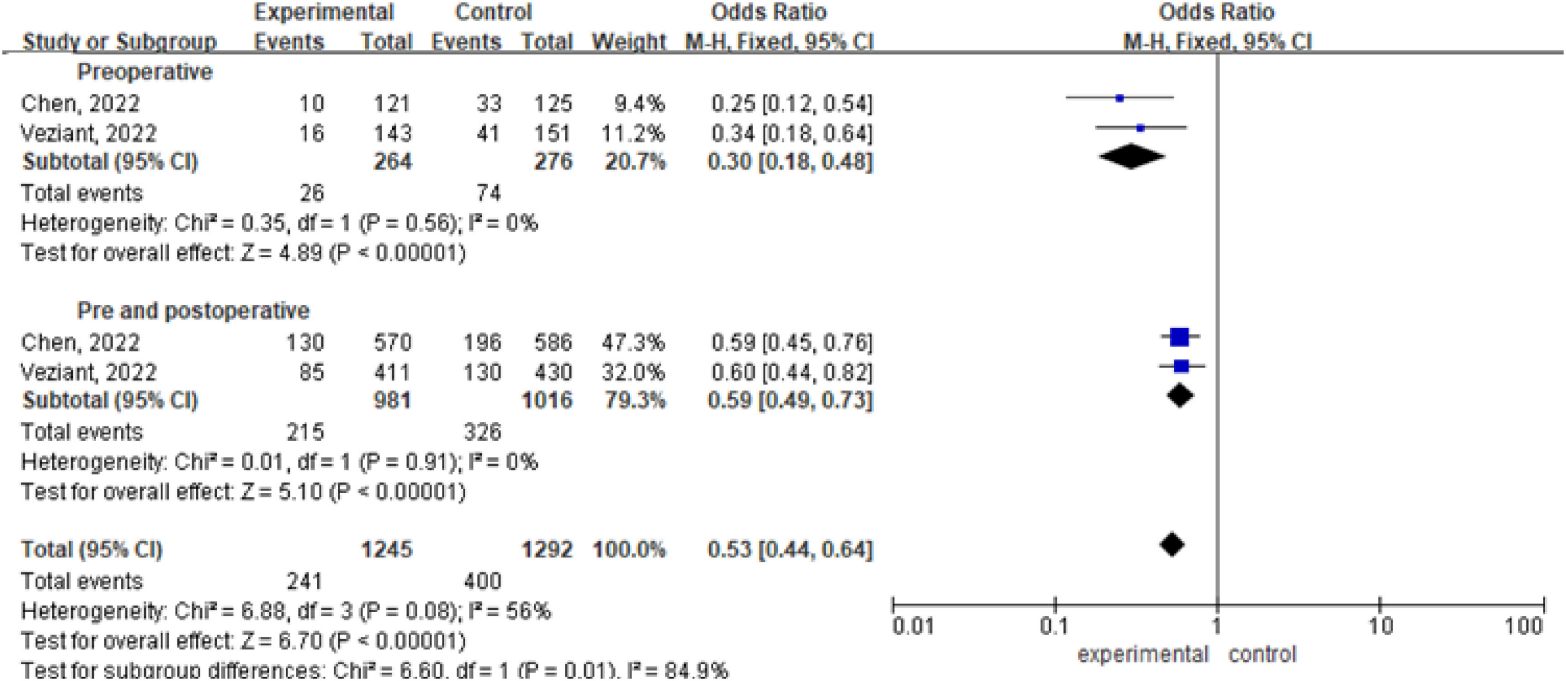
Forest plot of overall infection incidence with preoperative/preoperative and postoperative administration of probiotics/synbiotics.

There are 2 meta-analyses, with a total of 2537 patients included (Chen et al., 2022; Veziant et al., 2022). Analysis via a fixed effects model revealed statistically significant differences within the preoperative, pre and postoperative probiotic/synbiotic and control groups (OR 0.30, 95% CI: 0.18, 0.48; *P* < 0.00001, I^2^ = 0%), (OR 0.59, 95% CI: 0.49, 0.73; *P* < 0.00001, I^2^ = 0%). The subgroup difference was significant (OR 0.53, 95% CI: 0.44, 0.64, *P* = 0.01, I^2^ = 56%) (Figure 3).

**FIGURE 4:**
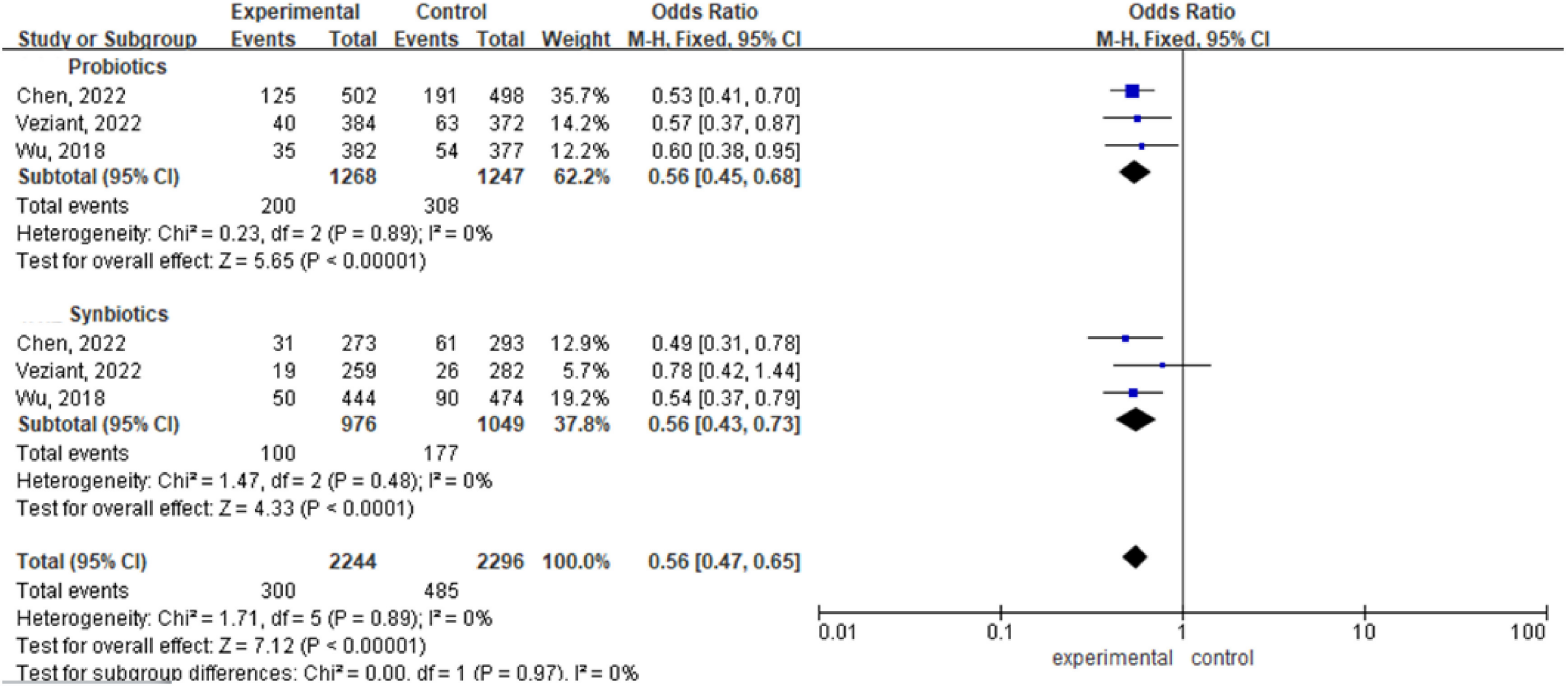
Forest plot comparing the effects of perioperative supplementation with probiotics or synbiotics on surgical site infections.

There are three meta-analyses (Chen et al., 2022; Veziant et al., 2022; Wu et al., 2018) including a total of 4540 patients. Analysis via a fixed effects model revealed statistically significant differences between the probiotic/synbiotic group and the control group during the perioperative period (OR 0.56, 95% CI: 0.45, 0.68; *P* < 0.00001, I^2^ = 0%) (OR 0.56, 95% CI: 0.43, 0.73; *P* < 0. 00001, I^2^ = 0%). The subgroup difference between probiotics and synbiotics was not significant (OR 0.56, 95% CI: 0.47, 0.65, *P* = 0.97, I^2^ = 0%) (Figure 4).

**FIGURE 5:**
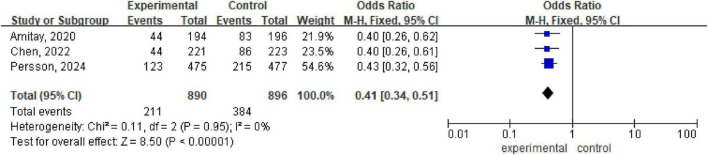
Forest plot of diarrhea incidence.

There are three meta-analyses (Amitay et al., 2020; Chen et al., 2022; Persson et al., 2024) with a total of 1786 patients included. Analysis via a fixed effects model revealed statistically significant differences between the probiotic/synbiotic group and the control group (OR 0.41, 95% CI: 0.34, 0.51; *P* < 0.00001, I^2^ = 0%) (Figure 5).

Among other results, the administration of probiotics/synbiotics in perioperative CRC patients improves gastrointestinal discomfort (Persson et al., 2024), shortens the time to first postoperative bowel movement (Amitay et al., 2020), and improves postoperative quality of life (An et al., 2022; Wu et al., 2018). The administration of single versus multiple probiotic regimens had no significant effect on surgical site infections or overall infections complications (Chen et al., 2024; Veziant et al., 2022). An et al. (2022) reported that the use of probiotics/synbiotics was not associated with any probiotic-related adverse events; certain probiotic strains may exploit the impaired immune system function of cancer patients, transforming into opportunistic pathogens and causing life-threatening pneumonia, endocarditis, and sepsis. Furthermore, the uncontrolled overuse of probiotics may lead to the transfer of plasmid-mediated antibiotic resistance to intestinal pathogens. These factors may hinder the target population from deriving benefits from probiotics/synbiotics administration (Kothari et al., 2019).

We calculated and assessed the overlap in the original studies using corrected coverage area (CCA) calculations based on current guidelines for overlap issues (Hennessy and Johnson, 2020). Our analysis showed that CCA = 0.0123 (<0.15), indicating that the overlap between the original RCTs in different meta-analyses was negligible. Details of the calculation are provided in the Supplementary Appendix 2.

## Discussion

Our review included 11 meta-analyses that evaluated the effects of perioperative probiotic/synbiotic supplementation in patients undergoing resection for CRC. In order to further evaluate the efficacy, we did an umbrella review in this study.

In our review, we found that perioperative CRC probiotic/synbiotic supplementation was beneficial in reducing the incidence of overall infection incidence, surgical site infection, urinary tract infection and pneumonia, and we also found that there was no difference in the reduction in surgical site infection between preoperative or preoperative and postoperative probiotic/synbiotic supplementation, and that there were no differences in the reduction in overall infection incidence with probiotics or synbiotics. Fortunately, there was little heterogeneity in our results, which may be because we were limited by the number of included trials and did not perform subgroup analyses of probiotic/symbiotic strains, dosage, time of administration, route of administration, and duration of administration to explore the effects on CRC patients. In particular, we combined CRC patients who used probiotics/synbiotic into one experimental group, which may have biased the results. Other potential sources of heterogeneity could be the demographic and clinical characteristics of CRC patients in different trials, such as age, treatment stage and surgical approach. These difficulties make it difficult to develop optimal protocols, which may be important factors affecting clinical practice and uptake.

An interesting finding of our study was that probiotic/synbiotic supplementation was better for “non-surgical” infection complications [i.e., pneumonia infection (OR = 0.34) and urinary tract infection (OR = 0.35)] than for SSI (OR = 0.60) and overall infection (OR = 0.50). This finding was first proposed by Liu et al. (2017). This leads us to hypothesize that this difference in effect size can prove that the gut microbiota has some association with lung and urinary tract infections, which will provide new ideas for the clinical prevention and treatment of lung and urinary tract infections (Neugent et al., 2020).

Previous studies have shown that probiotic/synbiotic supplementation has great potential to correct intestinal microbial dysbiosis, which may have significant clinical benefits for CRC patients, such as inhibiting the colonization of intestinal microbial pathogens (Richards et al., 2024), improving intestinal barrier integrity (Macharia et al., 2023), modulating host immune responses (Li et al., 2024), reducing therapeutic toxicity (Alam et al., 2022), and even inducing tumor cell apoptosis (Salek et al., 2024), thereby enhancing therapeutic efficacy (Xu et al., 2023). The metabolic metabolites of *Lactobacillus*, such as antioxidants such as glutathione, superoxide dismutase and catalase, have unique efficacy in alleviating intestinal inflammation and inhibiting the expression of tumor-specific proteins and polyamine components (Colbert et al., 2023; Garbacz, 2022; Wu et al., 2021). Bifidobacteria can activate immune cells such as macrophages, NK cells and B lymphocytes and promote their release of IL-1, IL-6 and TNF-α, thereby exerting indirect antitumor effects (Sivan et al., 2015). Probiotics or synbiotics, with their ability to reverse microbiota imbalances and modulate intestinal immune responses, are promising interventions for the comprehensive management of CRC. However, the American Society of Health-System Pharmacists does not recommend the use of probiotics in surgical patients (Bratzler et al., 2013) owing to limited data and poor implementation. More clinical trials and evidence are needed to support the promotion of the administration of probiotics or synbiotics in perioperative CRC patients (Yao et al., 2023).

### Limitations

(1) The included meta-analyses differed in their inclusion/exclusion criteria, which may have affected the synthesis of the results. (2) Generally low quality of the included reviews and potential overlap bias. (3) Due to limitations in the number of included studies, this prevented us from determining the specific use of probiotics/synbiotics.

## Conclusion

According to the results of our analyses, perioperative probiotic/synbiotic supplementation in CRC patients is associated with a reduced incidence of overall infections, surgical site infections, urinary tract infections, pneumonia infections, and diarrhea.

## Data Availability

The original contributions presented in this study are included in this article/Supplementary material, further inquiries can be directed to the corresponding author.
