# Supplementary material for: Probiotics/synbiotics supplementation reduce the infection incidence in patients undergoing resection for colorectal cancer: an umbrella review
Source: Front Microbiol. 2025 Sep 11;16:1635409. doi: 10.3389/fmicb.2025.1635409 (PMC12461257; doi:10.3389/fmicb.2025.1635409)

Appendix 1. Search strategy for PubMed.

| **Search** | **Query** |
| --- | --- |
| **#1** | (((("Probiotics"[Mesh]) OR (Probiotic*[Title/Abstract])) OR (prebiotic*[Title/Abstract])) OR (synbiotic*[Title/Abstract])) OR ("Probiotics"[Mesh]) OR ("Synbiotics"[Mesh]) |
| **#2** | **((((((colorectal cancer[Title/Abstract]) OR (rectal*[Title/Abstract])) OR (colon*[Title/Abstract])) OR (Colon* Cancer[Title/Abstract])) OR (Rectal* Neoplasms[Title/Abstract])) OR (Colon* Neoplasm[Title/Abstract])) OR ("Colorectal Neoplasms"[Mesh])** |
| **#3** | (((meta-analysis [Title/Abstract]) OR (systematic review [Title/Abstract])) OR (("Systematic Reviews as Topic"[Mesh]) OR (meta-analysis [MeSH Terms]))) |
| **#4** | #1 AND #2 AND #3 |

Appendix 1. Corrected coverage area


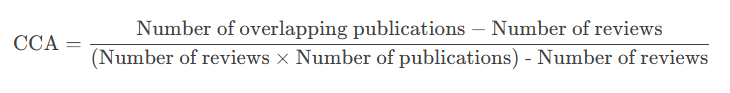


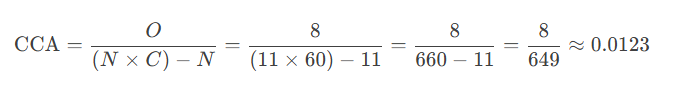

Supplement: Supplementary file 1 [file Supplementary_file_1.docx]
